# Mediterranean Seaweed Polysaccharides: Insight into Chemical Structures, Applications, and Structure/Application Correlations

**DOI:** 10.3390/md24010011

**Published:** 2025-12-24

**Authors:** Silvia Fanina, Angela Casillo, Maria Michela Corsaro

**Affiliations:** 1 Department of Chemical Sciences, University of Naples Federico II, Via Cintia 4, 80126 Napoli, Italy; silvia.fanina@unina.it (S.F.); angela.casillo@unina.it (A.C.); 2 National Biodiversity Future Center (NBFC), 90133 Palermo, Italy

**Keywords:** polysaccharides, Mediterranean Sea, biological activities, extraction and purification methodologies, structure-activity relationship, macroalgae

## Abstract

Although extensive research has been conducted on algal polysaccharides worldwide, Mediterranean species remain comparatively understudied, despite the region’s rich biodiversity and the presence of several endemic taxa with promising biotechnological potential. This review provides an overview of the major polysaccharides isolated from Mediterranean macroalgae, highlighting their structural features and bioactivities, as well as potential structure-activity relationships. Furthermore, the extraction and purification strategies used to isolate these biomolecules, ranging from conventional chemical approaches to emerging green technologies, were overlooked. Overall, the growing evidence of potent biological activities, combined with advances in sustainable extraction, underscores the significant potential of Mediterranean macroalgal polysaccharides as valuable resources unlocking new opportunities for their application in pharmaceutical, cosmetic, biomedical, and biotechnology fields.

## 1. Introduction

The Mediterranean Sea is considered one of the main biodiversity hotspots in the world, as it harbours a conspicuous number of marine species, especially those considered endemic [1]. For these reasons, the Mediterranean is defined as a unique environmental basin. It has been reported that 1124 species of seaweed live in the Mediterranean, and only 20% are endemic [2]. Furthermore, 800 seaweed taxa are located just in Italy and, among them, brown algae such as *Saccorhiza polyschides*, *Laminaria rodriguezii*, and *Fucus virsoides* are considered paleoendemisms [3].

Seaweed plays a pivotal role in the EU Bioeconomy Strategy, which aims to implement a circular bioeconomy throughout Europe, and in the EU Blue Growth Strategy, which seeks to generate employment in coastal regions and utilise innovative biotechnologies to harness resources from the sea. Algae are already widely used in Asia as food, and in recent years, they have slowly entered the Western diet since the discovery of their nutritional content and the need to look for alternative and sustainable food sources [4,5,6]. Seaweed is also largely collected for its extracts’ biotechnological, nutraceutical, and biomedical applications. Some species, such as *Laminaria digitata* and *Laminaria hyperborea*, are used as a source of alginate [6].

Seaweed plays an important ecological role in the marine environment. They are considered the primary food sources for marine herbivores. Furthermore, they are bioconstructor species, providing many diversified micro-habitats used by other species as nurseries and feeding grounds. The ability of marine seaweed to colonise different environments is reflected in the variety of high-value biologically active molecules they produce, i.e., terpenoids, carotenoids, alkaloids, diterpenes, peptides, and polysaccharides.

Polysaccharides derived from macroalgae fulfil critical functions within marine ecosystems, contributing significantly to ecological balance and ecosystem services. Seaweed constitutes a fundamental component of the marine food web, supplying essential polysaccharide-derived carbon sources for herbivores [7]. Additionally, they play a pivotal role in nutrient cycling through the utilisation of anionic polysaccharides, which function as biological filters by binding dissolved nutrients such as nitrogen and phosphorus, thereby enhancing water quality in coastal ecosystems [8]. Furthermore, seaweed facilitates microbial modulation via sulfated polysaccharides, which influence marine microbial communities by promoting beneficial bacteria while inhibiting pathogenic species through prebiotic effects [9]. The Mediterranean Sea, impacted by climate change, benefits from the remarkable environmental adaptation and resilience demonstrated by seaweed. These species act as carbon sequesters due to their polysaccharide-rich cell walls, forming long-term carbon sinks that mitigate ocean acidification [10]. Moreover, the carbon storage capacity of algal polysaccharides, particularly in brown seaweeds, contributes to mitigating the impacts of climate change in coastal zones [10,11]. Lastly, cell wall polysaccharides (CWPs) are one of the main cell wall components, reaching 75% of the dry weight, and they are used by the cell for photosynthetic storage or act as osmoprotectants [12].

The primary structure of CWPs varies across different genera. Alginate and fucoidans are found in brown seaweeds, while red seaweeds contain agar and carrageenan; green seaweeds, on the other hand, are known for their high content of ulvans.

High production of polysaccharides in seaweed cell walls is multifold: providing mechanical shear resistance [13], increasing fronds’ flexibility [14], facilitating cell–cell adhesion [13], and interacting with dynamic marine environments. The quantity and composition of these polysaccharides vary across different genera and are influenced by several factors, including the seasonal changes, growth stage of the algae, harvest, and maturity. For instance, in certain brown algae, the fucoidan content reaches its peak during reproductive phases [15]. Furthermore, as brown seaweeds mature, they exhibit increased alginate stiffness, characterized by a higher proportion of guluronic acid (G blocks), which enhances their resistance to wave action [16]. Steinhagen et al., [17] demonstrated that the total carbohydrate content in the green algae *Ulva fenestrata* increased by 29% during the summer compared to the spring. Additionally, the sulfate content in the polysaccharides of red algae, such as *Osmunda* sp., reaches its peak in the summer, while monosaccharide ratios exhibit seasonal shifts. Overall, it appears that early spring harvests optimize the yield of proteins, phenolic compounds, and pigments, whereas late spring and summer harvests are more favorable for carbohydrate yields [17,18].

Variations in polysaccharides may also arise due to abiotic factors such as salinity and temperature, as well as biotic factors like pathogens. [19,20]. These polysaccharides function as a barrier against such factors, offering a protective layer that aids in preventing infections and mitigating stress conditions. Ginneken [21] demonstrated well how salinity exercises a significant influence on the production, composition, and function of polysaccharides in seaweeds, thereby shaping their adaptive strategies to salt stress. One such strategy involves ion regulation, wherein sulfated polysaccharides form hydrogels that bind Na^+^ and Mg^2+^ cations, thereby reducing intracellular ion toxicity and maintaining osmotic balance. Furthermore, salt stress induces the production of reactive oxygen species (ROS); however, sulfated polysaccharides have been shown to activate antioxidant enzymes, such as catalase and peroxidases, to neutralize ROS and protect macromolecules, including lipids and DNA, from oxidative damage.

Algal polysaccharides are commonly known to be metabolically active but present a challenge when it is necessary to identify their structural features. This difficulty is influenced by various factors such as the genus of the algae, the specific location of the polysaccharide within the cell, the techniques used for extraction and post-extraction processing, and the potential secondary modifications to the monosaccharides, such as methylation and acetylation, which can lead to an infinite number of possibilities [22].

Mediterranean seaweeds exhibit potential as sources of polysaccharides, although the number of species already studied remains limited, with many endemic species being under-researched in comparison to global studies. This review aims to investigate the biodiversity of polysaccharides found in macroalgae in the Mediterranean Sea and assess the extent to which they have been explored and characterised to date. Furthermore, the review will address extraction techniques and the application of polysaccharides for industrial purposes. Finally, the structural features that influence the applications of these polymers will be evaluated.

## 2. Polysaccharide Distribution in Mediterranean Seaweeds

Seaweeds are broadly classified into three main groups, brown, red, and green, based on their pigmentation.

**Figure 1:**
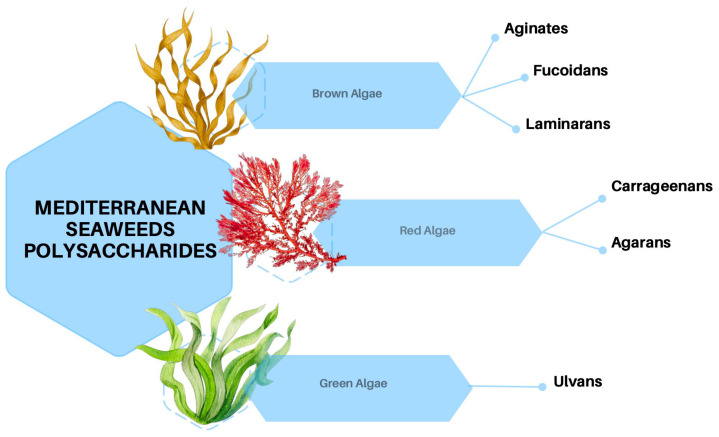
Polysaccharides produced by brown, red, and green seaweeds.

Brown algae (Phaeophyceae) predominantly consist of large, multicellular marine seaweeds, which are distributed across subtropical to colder coastal waters [23]. Their characteristic olive or light-golden brown coloration is attributed to the presence of the pigment fucoxanthin, in conjunction with chlorophylls *a* and *c* [24]. Morphologically, these algae exhibit a range of forms, from small filamentous structures to complex organizational architectures [25]. Analogous to terrestrial plants, the cells of brown algae are encased in a cell wall rich in polysaccharides [26]. The primary polysaccharides identified in brown algae include alginate, which is crucial for structural support by modulating mechanical properties [27]; fucoidan, a sulfated polysaccharide known for its bioactive properties [28]; and laminarin (also called laminaran), which mostly serves as storage [29] (Figure 1). Lastly, cellulose is present in minimal quantities and functions as a minor structural polymer [30].

**Figure 2:**
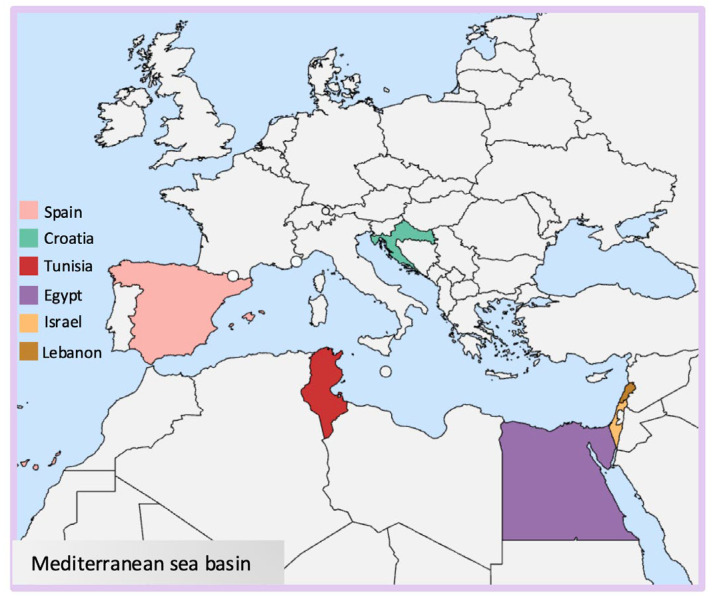
Map indicating the locations of seaweeds investigated for this study.

The brown algae hereby cited are mostly located in Tunisia, and a few species are found between Croatia and the Lebanese coast (Figure 2).

Red algae (Rhodophyta) are mostly marine algae commonly found in deeper waters. Their distinctive red color is due to phycoerythrin pigments combined with chlorophyll *a* and d, and they lack chlorophyll *b* [31]. Their cell walls generally contain sulfated galactans, which are generally classified as agarans, carrageenans, and porphyran [32] (Figure 1). These high molecular weight polysaccharides are widely used in the food industry and known for their rheological properties such as thickening and gelling agents [33]. The species object in this paper has been isolated mostly in Egyptian coasts and other species between Tunisia, Lebanon, and Israel (Figure 2).

Green algae are photosynthetic eukaryotes belonging to the phylum Chlorophyta and are also included in the broader group of Viridiplantae, where land plants can be found. Green macroalgae are being recognized as a source of unique polysaccharides, including ulvans (Figure 1), characterized by novel structures and interesting biological activities with different potential applications in medical, pharmaceutical, and biotechnological industries [34]. Species reported in this context are sampled mainly from Egyptian coasts, followed by Tunisia and Spain (Figure 2).

### 2.1. Brown Algae

#### 2.1.1. Alginates

**Figure 3:**
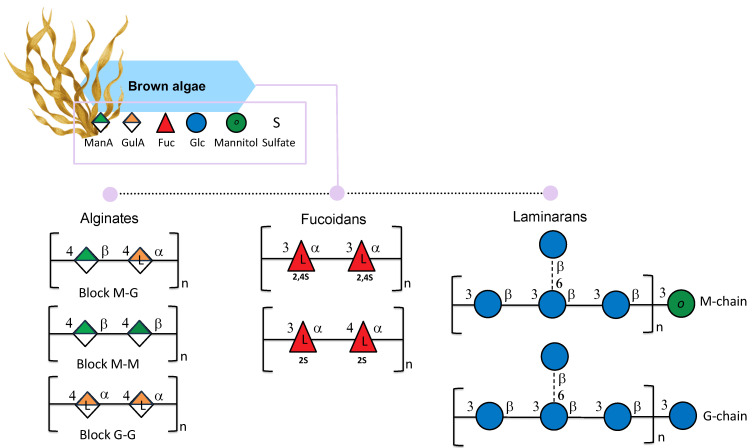
Structural variations of alginates, fucoidans, and laminarans isolated from brown seaweeds. All glycan symbols follow the symbol nomenclature for glycans (SNFG) guidelines.

Alginate is the salt of alginic acid, a polymer comprising β-D-mannuronic acid (M) and α-L-guluronic acid (G). The two monosaccharides are linked through (1,4) bonds to form the block molecular structure of homopolymers (poly-M or poly-G) and/or heteropolymers, composed of mixed and alternating sequences of residues G and M (Figure 3). The M/G ratio in alginates dictates the polymers’ physicochemical properties, and therefore their industrial applications [35]. Indeed, alginates with a ratio < 1, indicating a higher value of guluronic acids (G) than mannuronic acid (M), can form strong gels; a ratio > 1 indicates a low amount of guluronic acids (G) with the consequent production of softer and more elastic gels [35].

The M/G ratio values of Alginates from *C. compressa* showed a high content of FGG (fraction of alginate consisting of guluronic acid residues) homopolyguluronic blocks, which are reported to be able to form a solid and rigid gel useful as a heavy metals’ adsorbent material for industrial applications [36]. Indeed, it has been reported that one of the main applications of alginates is the capability of the polysaccharide chains to cross-link through divalent cations, which shows the formation of an “egg box” gel structure. For their gel-forming capacity, alginates are particularly suitable in food applications since they can restructure the damage caused by high temperatures in meat products, fruits, and vegetables [37]. Particularly important is the yield of the alginate extracted from *C. compressa* (21.65% *w*/*w*), which was revealed to be higher than that obtained with other species of *Cystoseira*, such as *C. sedoides* (11%) and *C. barbata* (9.9%) [36,38,39] (Table 1).

Another alginate, extracted from *Cystoseira sedoides* by Ammar et al., [38] showed elastic properties due to a higher amount of mannuronic acid blocks (M) with 63% and 37% of guluronic acid blocks (G). The molecule also showed gastroprotective effects against gastric ulcers. Interestingly, the alginates extracted had a total yield of 11%, and no further characterization was accomplished.

Faidi et al., isolated alginate from another brown seaweed *Padina pavonica* and reported its characterization [40]. With a yield of 28.7% and a molecular weight of 4440 KDa, the extracted alginate M/G ratio was 0.36, specifically composed of FG 0.74, FM 0.26, FGG 0.51, FMG (fraction of alginate consisting of heteropolymannuronic/glucuronic blocks) 0.23, FGM (fraction of alginate consisting of heteropolyguluronic/mannuronic blocks) 0.23, and FMM (fraction of alginate consisting of homopolymannuronic blocks) 0.03. The characteristic high molecular weight of this alginate and its M/G ratio can lead to a more viscous solution able to form gels that can be used for drug-delivery systems in the form of nanoparticles [40]. The authors compared the extracted alginate to the commercial one by using Attenuated Total Reflection (ATR) FT-IR Spectroscopy, ^1^H, and ^13^C monodimensional nuclear magnetic resonance (NMR).

ATR FTIR analysis of *P. pavonica* alginate showed characteristic peaks, similar to those of the commercial. In addition, ^1^H NMR spectra from both alginates also showed similar profiles and signals but with different intensities for some of them, pointing out a few differences in structure. The assignment of proton and carbon signal chemical shifts was done by highlighting different distributions of blocks of GG, MM, or GM. *Padina pavonica* was also collected in different months (April–July) on the shores of the Mediterranean Sea in Lebanon. After the extraction of the defatted algae biomass, the corresponding alginates have been isolated. Since it has been demonstrated that the season influences the content of M/G, the alginates were found to show different gel-forming abilities. The highest guluronic content polysaccharide was found to possess the highest viscosity [41].

Abid et al. also reported alginate contents of the brown algae *Dictyopteris membranaceae* and *Padina pavonica* [58], but only uronic contents were disclosed for both the polymers.

#### 2.1.2. Fucoidans

Fucans are polysaccharides of fucose backbones mainly found in brown algae cell walls [59]. Among fucans, fucoidan is a fucan-sulfated polysaccharide characterized by two slightly different backbones: one composed of (1→3)-linked α-L-fucopyranose residues and the other with a central chain composed of repeating (1→3)- and (1→4)-linked α-L-fucopyranose residues (Figure 3); the first one is most common in *Laminaria* species and the second in *Fucus* species. In addition, the sulfated groups can be found at the C2 and C4 positions [60]. Nonetheless, it is reported that fucans can be characterized by a more complex structure and can contain a small amount of other monosaccharides such as mannose, glucose, galactose, xylose, and uronic acids [61]. The amount and the structure of fucoidans in brown algae can vary due to season, reproduction, environmental factors, and tissue positions, as Bruhn et al., reported in *Laminaria* species [62]. Moreover, the extraction techniques used can lead to different forms of fucans [63]. The degree of sulfation and the molecular weight of fucoidan can determine the molecule’s biological activity [64].

Since fucoidans have been proven to display many biological activities, such as antiviral and immunomodulant, their use in various sectors has been explored [65]. Some applications of fucoidans are in the food industry. For example, fish aquafeed containing fucoidans has been demonstrated to have a role in growth-promoting in fish and shrimp [66].

*Cystoseira barbata* and *Fucus virsoides* are considered dominant brown algae in the Mediterranean Sea [66,67]. Extracted crude polysaccharides had a total yield of 6.37% for *C. barbata* (harvested from the coastal region of Zadar, Croatia) with a sulfate content of 34.8% and 15.40% for *F. virsoides* (harvested from the southwest coast of Novigrad Sea, Croatia) with a sulfate content of 25.6%, respectively. This amount was obtained using microwave-assisted extraction (MAE) and pressurized liquid extraction (PLE) as extraction techniques. The fucoidan extracted from *F. virsoides* had 58.55% fucose content while *C. barbata* reached 26.13% [42]. According to their molecular weight, fucoidans are classified as: low-molecular-weight fucoidans (<10 kDa), medium-molecular-weight fucoidans (10–10,000 kDa), and high-molecular-weight fucoidans (>10,000 kDa). The molecular weight of fucoidans from *F. virsoides* was between 522 and 890 kDa and from *C. barbata* was between 766 and 1250 kDa. Both species showed antioxidant activities [42]. Different sulfate contents in both seaweeds were observed depending on the temperature and solvent used for extraction [42]. No structure hypothesis has been made for these two fucoidans. Sellimi et al. [67] also reported on a galactofucan extracted from the seaweed harvested from Kerkennah Island, Tunisia. In this case, the sulfate content was 22.51%. The polymer was mainly constituted by α-(1→3) Fuc and (1→3) Gal, with branching points at position 2 of the fucose. The Fuc/Gal ratio for this species was 1.3.

The polysaccharide extracted from *Cystoseira compressa* (harvested from Kerkennah island, Tunisia) [36] has been reported as a sulfated heterogalactofucan (sulfate content 14.65%) composed primarily of fucose and galactose, with smaller amounts of glucose, xylose, and glucuronic acid. Its backbone is composed of α-(1→3) and α-(1→4)-linked L-Fucp, which is branched due to 3,4-α-L-fucose units (13.2%). The branches end with terminal α-L-Fucp, terminal β-D-Galp, β-D-Galp-(1→3)-α-L-Fucp and β-D-Galp-(1→4)-α-L-Fucp. The Fucp/Galp ratio in this species was reported as 2.32 [36].

The Fucoidan extracted from *Cystoseira sedoides* had a total yield of 4.2% and was highly sulfated (15.5%) with 51.3% of total sugars, 7.6% of uronic acids, and 17.6% as amount of fucose. The molecule showed antioxidant activities due to the presence of sulfated and acetyl groups [38]. Fucoidans isolated by Ammar et al., from *C. compressa*, *C. sedoides* and *C. crinita* showed no significant differences concerning the extraction yield (3%), and fucose was the main monosaccharide, ranging from 43 to 61%. The amount of sulfate was around 16% for all three species and was confirmed by FTIR analysis with bands at 1255–1240 cm^−1^, indicating the S=O stretching, and at 820 cm^−1^, indicating the presence of sulfate groups at positions 2 and/or 3. In terms of molecular weight, fucoidans extracted from the three *Cystoseira* species showed some slight differences: *C. sedoides* had a molecular weight of 642 KDa, *C. compressa* 545 KDa, and lastly *C. crinita* 339 KDa [43]. All species showed positive antioxidant, antiradical, and gastroprotective activities. In Ben Gara et al., [44], it has been explained++ that polysaccharide from *C. crinita* was mainly composed of arabinose (16%), followed by galactose (8.98%), allose (7.5%), altrose (7%), mannose (6.9%), and traces of arabinofuranose, glucosamine, and glucose. The sulfated polysaccharide showed potential therapeutic applications in hyperlipidemia and other liver lipid dysfunctions.

Research has demonstrated that the sulfation content and overall composition of fucoidans can fluctuate with seasonal changes. Benslima et al., [45], examining the seasonal chemical variation of fucoidans in *Cystoseira schiffneri* revealed that the highest yield of fucoidan was observed in December. This increase may be attributed to abiotic stressors such as reduced temperatures, diminished light exposure, and decreased salinity, thereby illustrating the influence of environmental factors on polysaccharide content. Conversely, the lowest yield was recorded in July, coinciding with the reproductive phase of the algae’s life cycle. Given that fucoidans are reported to be essential during sporulation periods since they are released immediately before the spores [45], this may account for the reduced yield observed during this period. The sulfate content had the opposite trend since it was the highest in July and the lowest in December. Many authors reported that most of the activity of these macromolecules resides in the sulfate content [68,69], and therefore, in this case, the summer season is considered the most appropriate to extract polysaccharides with the highest sulfate content. Regarding monosaccharides, *Cystoseira schiffneri* fucoidan was composed mainly of mannose, fucose, galactose, and uronic acids, with lower amounts of xylose, arabinose, and mannitol recorded during April and June. The molecular weight of the extracted polysaccharides was around 26 KDa in June and around 4 KDa in December. Finally, the authors reported an increase in antioxidant activity in April due to the molecular weight, monosaccharide distribution, and sulfate group type.

The content of fucoidans extracted from *Padina pavonica* sampled on the Lebanese coast was analysed by Mencshova et al. [41] from April to July, confirming a season-changing trend. Fucoidan content was pretty low during May (0.09%), with an increase in June (0.23%) and the highest point in July (12.5%). Instead, variable sulfation of the polysaccharides was observed, ranging from 18.5% to 0.1%. In this contest, fucoidan was tested for antitumor activity, and the highest activity was shown in June with 54% growth inhibition of human melanoma cell colonies.

#### 2.1.3. Laminaran

Laminaran (also known as laminarin) is a polysaccharide built up of β-D-1,3-glucose branched in some cases at O-6-positions and with β-D-1,6-glucose intrachain-links. In addition, glucose (G-chains) or mannitol (M-chains) residues can be found in different ratios on reducing ends (Figure 3) [70,71]. Laminaran can be used for many applications since it is involved in many biological activities, including prebiotic, antioxidant [72], bone regeneration [73], anticoagulant, hypocholesterolemic, and antimutagenic activity [74].

The backbone of laminaran extracted from *Cystoseira barbata* by Sellimi et al., [39] was organized as follows: 70% of 3-linked β-D-glucopyranose units, with 15% of linkages in O-3 and O-6 positions, and only 4% of glucopyranose linkages were in O-6 positions. In addition, the isolated laminaran tested positive for antioxidant, antibacterial, and wound-healing activities.

*Padina pavonica* laminaran content was very low [41]. In particular, the samples collected between April, May, and July contained pure glucans, whereas the laminaran was absent from the sample collected in June.

#### 2.1.4. Other Polysaccharides

A heteropolysaccharide was probably the component of the extract from the brown algae *Dictyopteris membranacea* [75]. The alga was extracted with water at 25 °C and 80 °C, showing differences in terms of yield, which was 2.14% and 3.89%, respectively. After hydrolysis, the total monosaccharides was found to be 87.3% and 67.8% for low and high temperatures, respectively. Furthermore, the HPLC (high-performance liquid chromatography) allowed the identification of seven monosaccharides at 25 °C and nine monosaccharides at 80 °C. *D. membranacea* polysaccharides were also tested for antimicrobial, antitumor, and anticoagulant activities, and the two extracts showed no significant differences, resulting in positive antimicrobial, antitumor, and anticoagulant activities [75].

### 2.2. Red Algae

#### 2.2.1. Carrageenans

**Figure 4:**
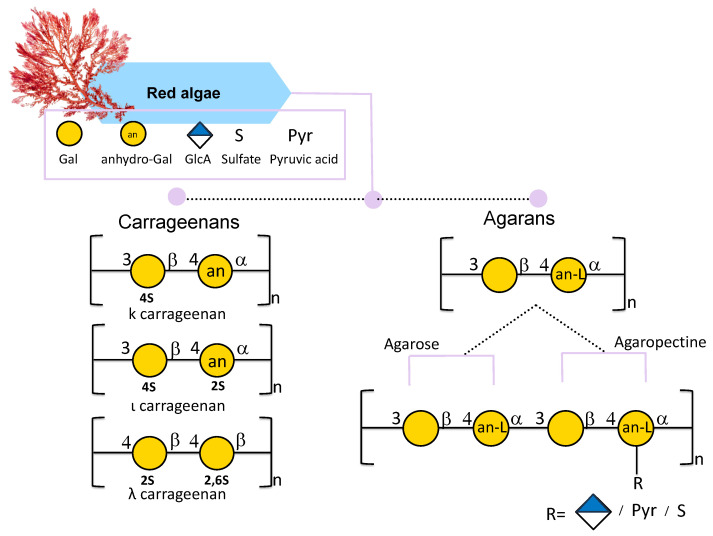
Structural variations of carrageenans and agarans from red seaweeds. All glycan symbols follow the symbol nomenclature for glycans (SNFG) guidelines.

Carrageenans extracted from red seaweeds are linear sulfated D-galactans consisting of a disaccharide repeating unit of 3-linked β-D-galactopyranose called G-units and 4-linked α-D-galactopyranose called D-units, or 3,6-anhydro-α-D-galactopyranose called DA-units (Figure 4) [46]. After extraction, carrageenan appears translucent from white to yellowish brown color; it is odorless and tasteless and can be sticky and slippery [76]. When it comes to applications, it is already largely used in the food industry thanks to its non-toxic, thickening, gelling, and emulsifying properties [77].

Carrageenans are traditionally referred to as Iota (ι)-, Kappa (κ)-, Lambda (λ)-, Mu (μ)-, Nu (ν)-, and Theta (θ)-carrageenans [77]. This nomenclature depends on their chemical structure and on commercial production. Among all κ, ι, and λ forms, differing in the position of the sulfate group, are more industrially relevant carrageenans (Figure 4) [65]. The κ carrageenan structure, mostly found in the red seaweed *Kappaphycus alvarezii*, is composed of a disaccharide repeating unit of 3-linked β-D-galactose 4-sulfate and 4-linked 3,6-anydro-α-D-galactose (AnGal) units (Figure 4) [78,79]. The ι-carrageenan differs from the previous one in the presence of an additional sulfate group in position 2 of the AnGal residue (Figure 4). It has been reported that an additional sulfate makes the ι-carrageenan more homogeneous and flexible [80]. The λ-carrageenan has an additional sulfate group per disaccharide unit at the C6 position of the 4-Gal residue and lacks the AnGal 3,6-anhydride bridge on the 4-linked residues [76].

Ismail & Amer. extracted and characterized polysaccharides from *Corallina officinalis* and *Pterocladia capillacea* with a yield of 37% and 43%, respectively [81]. The sulfate content was 3.2% in *C. officinalis* polysaccharides and 1.5% in *P. capillacea.* Through FTIR and ^1^H NMR analyses, uronic acids and ester sulfates were detected in both algae polysaccharides. Taking into account all the main information, the presence of agarocolloids and galactans was deduced in both cases. In the case of *P. capillacea*, the presence of 3,6-anhydrobridge was also detected in the FTIR spectrum [81].

Abou Zeid et al. [75] also sampled and extracted a polysaccharide from the red alga *Pterocladia capillacea* using a room temperature (25 °C) and hot water (80 °C) extraction, with a yield of 2.87% and 6.46%, respectively, proving how the extraction techniques can influence not only the amount of polysaccharide that can be extracted but also its conformation. In terms of sugars at 25 °C, galactose (25.36%), glucose (20.59%), arabinose (12.61%), and fructose (9.17%) were detected, while at 80 °C, galactose (17.81%), glucose (16.99%), fructose (9.38%), and mannose (7.37%) were the major sugars. The polysaccharide was tested for antimicrobial, antitumor, and anticoagulant properties, and no differences in the activities of the two extractions were detected; both polysaccharides showed positive results for all three activities.

#### 2.2.2. Agarans

Agar is a linear water-soluble hydrocolloid galactan with a gel-forming ability [47] mostly present in red algae cell walls [82]. It is composed of a disaccharide of alternating (1,3)-linked β-D-galactose and (1,4)-linked 3,6-anhydro-α-L-galactose. Agar can be constituted by agarose and agaropectin. Agarose is a linear and neutral polysaccharide composed of β-D-galactose and 4-linked 3,6-anhydro-α-L-galactose (Figure 4) [83]. Instead, agaropectin is an acid polysaccharide since agarobiose can be linked to sulfate groups, pyruvic acid, and D-glucuronic acid (Figure 4). It is reported that highly sulfated agar is mainly found in *Gracilariales* and in the *Pterogladia* species [84] and, compared to carrageenan, is less sulfated [85].

Mettwally et al. [86] extracted and purified a sulfated polysaccharide mainly composed of galactose from the red algae *Grateloupia gibbesii* Harvey. NMR studies allowed the detection of the presence of 3-linked-β-D-galactose and α-L-galactose residues. Other signals showed the presence of 3,6 anhydro-L-galactose and 4-O-linked α-L-galactose-6-sulfate units, which are considered agarose precursors. FT-IR analysis revealed the absence of carrageenan and the presence of potential agarocolloids. The authors concluded that *Grateloupia gibbesii* contained an agar-type polymer as already observed for *Grateloupia filicina* [48], *Gracilaria dura* and *Gracilaria crassissima* [87,88].

*Jania adhaerens* polysaccharide was extracted by 0.3 M NaOH solution [49] with a low yield (4.55%, *w*/*w*). This is probably due to the calcareous covers that characterize the Corallinales. The polysaccharide extracted was identified as sulfated xylogalactan, confirmed by 18,15% of 3,6-anhydrogalactose with a low presence of sulfate groups, suggesting a more driven natural feature of the polysaccharide. Monosaccharide composition showed a higher amount of Galactose (62,3%), Glucose (20%), Xylose (15%), and a lower amount of GlcA (2%) with a ratio of Gal/Xyl of 4.05, which is a variable aspect in corallines. ^1^H/^13^C NMR spectra revealed agar-like xylogalactan with a repeating backbone of →3)-β-D-Galp-(1→4)-α-L-Galp-(1→ and →3)-β-D-Galp-(1→4)-3,6-α-L-AnGalp-(1→ substituted at various positions by β-xylosyl residues, methoxyl and/or sulfate groups. The xylogalactan isolated from *J. adhaerens* shares these structural features with other similar polymer isolated from *Corallina officinalis* [50], *Joculator maximus* [89], and *Jania rubens* [90], but with less substitution at O-6 position of the (1,3)-β-D-Gal galactose units by sulfate or methoxyl groups. Instead, these groups can be found at O-2 and O-3 of (1,4)-α-L-Gal units [49]. As coralline polysaccharides are used for their hydrocolloid properties, the authors have hypothesized that *J. adhaerens* can be a new source of agaroids.

The amount and the variation of galactans in red algae vary due to seasonal changes, species, and harvest time, as demonstrated by Lunkes et al. [91] by extracting polysaccharides from red seaweed *Galaxaulra rugosa*, *Tricleocarpa fragilis*, *Liagora viscida*, *Osmande dechybrida*, and *Palisada perforata*. Among them, the highest percentage of carbohydrates was registered in *O. dechybrida* with 76.3% and the lowest in *G. rugosa* with 34.8%. The extraction yield was highest for *O.dechybrida* (14.32%), followed by *L. viscida* (10.9%), *P. perfosata* (7.5%), *G. rugosa* (5.1%) and lowest in *T. fragilis* (4.03%), proving once again how the extraction techniques and the type of species can influence the amount of polysaccharides extracted. The sulfation degree was higher in *G. rugosa* (28.5%), *L. viscida* (25.6%), and *P. perfosata* (20.3%) but only 14,9% in *O. dechybrida* and 8.2% in *T. fragilis*. The amount of uronic acids did not show major differences; it was higher in *O. dechybrida* (5.4%) and lowest in *T. fragilis* (2.87%). All species were tested for antioxidant and antiproliferative activities, which were proven to rely on the sulfate content and uronic acid concentration of the galactan. *G. rugosa* polysaccharide showed the most antioxidant and antiproliferative activities among all.

### 2.3. Green Algae

#### Ulvans

**Figure 5:**
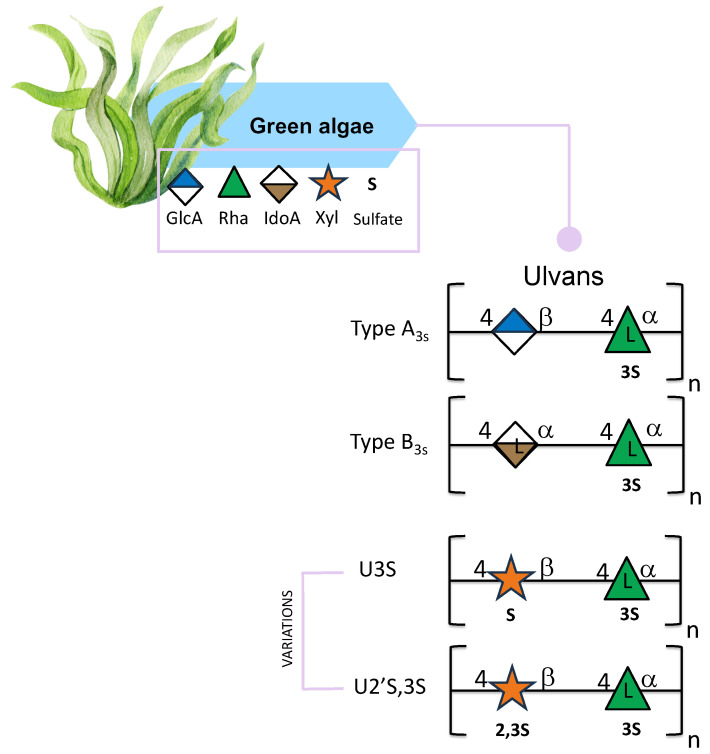
Structural variations of ulvans derived from green seaweed. All glycan symbols follow the symbol nomenclature for glycans (SNFG) guidelines.

Ulvans are considered polyanionic heteropolysaccharides mainly composed of rhamnose, glucuronic acid, and xylose. Ulvans contain sulfate groups and are the major water-soluble polysaccharides found in the order Ulvales [92,93]. The backbone of these polysaccharides is characterized by two types of disaccharide units: type A3s, which are composed of β-D-glucuronic acid (1,4)-linked to 3-sulfate-α-L-rhamnose, and type B3s, consisting of α-L-iduronic acid (a C-5 epimer of glucuronic acid) linked at position O-4 of a 3-sulfated α-L-rhamnose. The main difference between the two disaccharides is that one has glucuronic acid and the other has iduronic acid (Figure 5) [93]. Occasionally, minor repeat units have shown sulfated xylose replacing the uronic acid or glucuronic acid, forming ulvanobioses named U3S and U2′S,3S (Figure 5) [94]. U3S is composed of β-D-xylose linked to α 1,4 L-rhamnose-3-O-sulfate; U2′S,3S is composed of β-D-xylose-2,3-O-sulfate linked to α 1,4 L-rhamnose-3-O-sulfate (Figure 5) [95]. The composition of ulvans depends not only on the extraction techniques but also on the location, on the effect of external abiotic factors [96,97], and varies among species [96].

Green seaweeds of the *Ulva* genus are morphologically categorized into blade and filamentous species, each exhibiting distinct differences in ulvan polysaccharide composition. Specifically, blade species of *Ulva* are characterized by ulvan with elevated iduronic acid and rhamnose content, increased structural complexity [98], greater structural variability [99], and enhanced extraction efficiency [98] in comparison to filamentous species. Conversely, filamentous species such as *Ulva prolifera* demonstrate higher levels of xylose and glucose in their polysaccharides [51] and predominantly linear (1→4) linkages, which confer rigidity [51]. These differences are attributed to species-specific adaptations, ecological niches, and functional requirements, such as structural rigidity or flexibility [51,98,99].

Ulvans polysaccharides have also been investigated as fundamental components for hydrogel production, which have been evaluated for various applications. Notably, ulvan-based hydrogels have demonstrated efficacy as systems for controlled drug release, and as adsorbents for dyes or heavy metal ions [52,100]. Furthermore, due to their cytocompatibility and capacity to support cell proliferation, research indicates that ulvan hydrogels enhance the viability and growth of human dermal fibroblasts (HDFs), making them suitable for use as scaffolds in tissue engineering [98,100]. On this matter, Ghazy et al. [101] showed how ulvan extracted from the species *Ulva lactuca* (Egypt) could also be used for hydrogel purposes. More specifically, after obtaining an ulvan with a molecular weight of 43,513 g/mol they turned it into a film by dissolving it with a 1% acidic solution. The ulvan’s biofilm showed good absorbent abilities in removing blue methylene from an aqueous solution due to ionic interactions between the positive dye molecules and ulvan’s negatively charged carboxyl groups. Green algae are also a source of sulfated galactans, even if not as much as red algae; it is reported that they are more complex and heterogeneous in terms of structure. More specifically, they are highly branched and present more sulfated groups [53,69].

As mentioned before, extraction methods can influence polysaccharides in terms of monosaccharide composition and the arrangement of the molecule, showing different ulvan structures. Moawad et al. [54] extracted and characterized a sulfated polysaccharide from the green algae *Ulva fasciata* Delile by performing a two-step sequential extraction using distilled water, NaOH, HCl, and EDTA. The polysaccharide was analyzed at every step and at the end of the combined extraction techniques. Finally, they obtained a total extraction yield of 11.81% (*w*/*w*). The highest sulfate content was 21% using the HCl step. In terms of monosaccharide composition, rhamnose, galactose, and uronic acids were detected, and glucose and glucosamine were in traces. More specifically, the glucose concentration was higher in the HCl step since it is reported that using an acid medium for polysaccharides extraction leads to a higher glucose content [55]. ^1^H NMR spectra of each single extraction were obtained, and overlapping them showed complex polysaccharides, where the presence of O-acetyl groups was detected in fractions extracted with distilled water and EDTA. In terms of activities, the polysaccharide extracted using distilled water showed antimicrobial and antifouling activities; the higher antioxidant activity was shown by the polysaccharides extracted with HCl, which had the highest sulfate content. Lastly, the EDTA-extracted polysaccharide showed higher anti-inflammatory activity than the others, proving that the extraction methods can also influence the activity of the molecule. Moreover, Yaich et al. [102] proved that extraction techniques can influence the polysaccharides’ molecular weight, sulfate content, and compositions in terms of monosaccharides, which can be crucial for molecular activities. Even if FTIR and ^1^H NMR spectra showed the typical peaks of ulvan, the extraction procedures showed polysaccharides with different molecular weights ranging from 1240 to 2790 kDa and with a difference in terms of glucose or xylose signals. The maximum yield obtained was with the enzymatic extraction, whereas the lowest was obtained at 90 °C and pH 1.5, proving that at this temperature and a lower pH, the degradation of the polysaccharides can happen. Regarding sulfate content, the 90 °C extraction showed the lowest content, but no big differences were observed in the other extractions. The 90 °C acidic extract with a higher Mw (2790 kDa), regardless of the extraction procedures, showed the highest total antioxidant activity and was more effective against DPPH radical than the others. This proved that the Mw could influence the inhibitory effect on DPPH radical of ulvans. El Azm et al. employed enzymatic hydrolysis as an extraction technique for *Ulva lactuca* polysaccharides and anion exchange chromatography to analyze different fractions [56]. This approach aimed to determine whether the molecular weight could be associated with both the antioxidant properties and the antitumor activity of the hydrolyzed product, since the authors believe that the enzymatic method enhances the biological activity of the crude extract. It came out that the fractions with carbohydrate and sulfate comparable contents showed more antitumor activities. In terms of monosaccharides, fractions with comparable contents of rhamnose and glucuronic acid or glucose and arabinose were more active against tumor cells [56].

Hussein et al. extracted ulvans from two *Ulva* species: *Ulva lactuca* and *Ulva fasciata*, with a yield of 35% and 37%, respectively [57]. *U. lactuca* was mainly composed of rhamnose 34%, glucose and galactose 28%, xylose 23%, and arabinose 14%. *U. fasciata* showed a higher amount of rhamnose 48%, glucose and galactose 4%, and 10% of arabinose, with no presence of xylose. As for sulfate and uronic acid contents, both species showed a similar percentage. Viscosity measurements were performed for both polysaccharides, indicating pseudoplastic behaviour when the concentration of the polysaccharides was increased in the solutions. Pseudoplastic behavior arises from intra- and intermolecular association forming transitory network structure, which are strongly modulated by negatively charged groups such as carboxylates of uronic acids and sulfate esters, that participate in hydrogen bonding and ionic crosslink [103]. Both polysaccharides were tested for antioxidant activity and as a source of carbon in mixotrophic growth conditions for the microalga *Chlorella vulgaris*. Both polysaccharides showed positive antioxidant activity and were detected as a carbon source in promoting biomass accumulation in *C. vulgaris* growth. Maray et al. [104] sampled *U. lactuca* in May to demonstrate that during this season, the seaweed’s photosynthetic activity is enhanced, leading to a higher sugar content for growth [104]. The total sugar yield was 35% and the monosaccharide compositions, obtained by HPLC, pointed out galactose (19.2%), rhamnose (23.3%), glucose (35.21%), fructose (9.2%), and uronic acid content was 2.23%. The remaining 11.4% was attributed to melibiose, which is a disaccharide composed of galactose and glucose. FTIR analysis confirmed the polysaccharide as ulvan, and it was tested for anticancer activities, resulting in more cytotoxicity against the prostate cancer cell line but less against the lung carcinoma cell line. It also showed antiviral, antibacterial, and lower antioxidant activities.

Finally, the ulvan isolated from the green algae *Ulva rigida* sampled in Malaga, is reported to show antioxidant activity and is composed mainly of rhamnose, and less glucose, glucuronic acid, and xylose [105].

## 3. Methodologies

Macroalgal cell walls exhibit a complex structure, comprising polysaccharides embedded within proteoglycans, which collectively form an extracellular matrix. The intricate network of polysaccharides, interwoven with proteins and associated ions such as calcium and potassium, contributes to the arduous and time-intensive nature of the extraction process. The choice of a particular extraction technique is likely determined by the target polysaccharides, the species under investigation, and the desired polysaccharide extraction yield.

The extraction techniques employed for polysaccharides from macroalgae are numerous and can be categorized into groups: chemical techniques and advanced green techniques. In both cases, the extraction of polysaccharides is preceded by pretreatments to remove pigments, lipids, and other molecules. Furthermore, since seaweed hosts several epiphytes, washing and cleaning the algae is always performed to also remove debris, salts, pollutants, and sand.

### 3.1. Pre-Treatments

**Figure 6:**
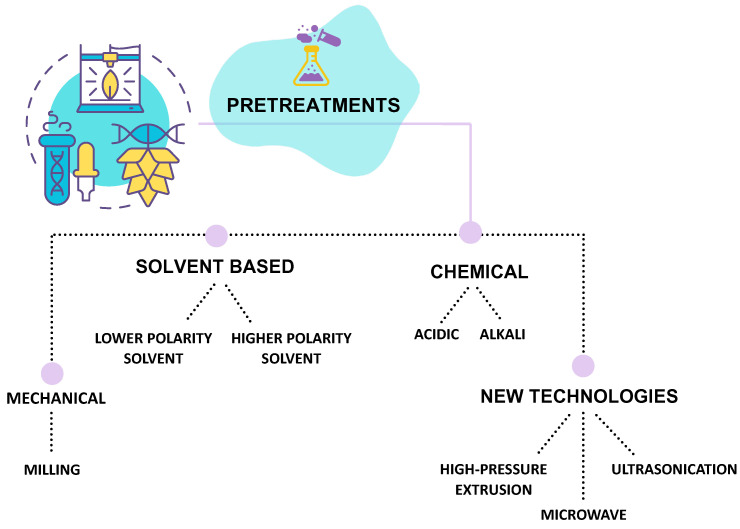
Schematic representation of pre-treatments used before seaweed polysaccharides extraction.

The applied pre-treatments can be mechanical, chemical, and solvent-based, and achieved using new technologies (Figure 6). Then the sample is usually freeze-dried or dried in an oven at 37 °C to mill it and obtain a powder to increase surface area for solvent penetration [7].

Typically, chloroform, petroleum ether, and dichloromethane, as lower-polarity solvents, are used to remove lipids, whereas those like acetone, methanol, and ethanol are used to remove pigments [36,38]. In some cases, a combination of methanol, chloroform, and water (4:2:1; *v*/*v*/*v*) can be performed to strip phenolic compounds and proteins [60,106,107]. In some cases, alkali pretreatment is used on red algae to improve gelling properties, and an acid treatment with hydrochloric acid is used for red algae to improve the solubility of sulfated polysaccharides present partially as calcium and magnesium salts in the cell wall [108]. In addition, to improve alginate extraction yield, formaldehyde is used as a pretreatment [109].

Finally, reported pre-treatments include milling, high-pressure extrusion, ultrasonication, and microwave procedures.

### 3.2. Extraction Techniques

**Figure 7:**
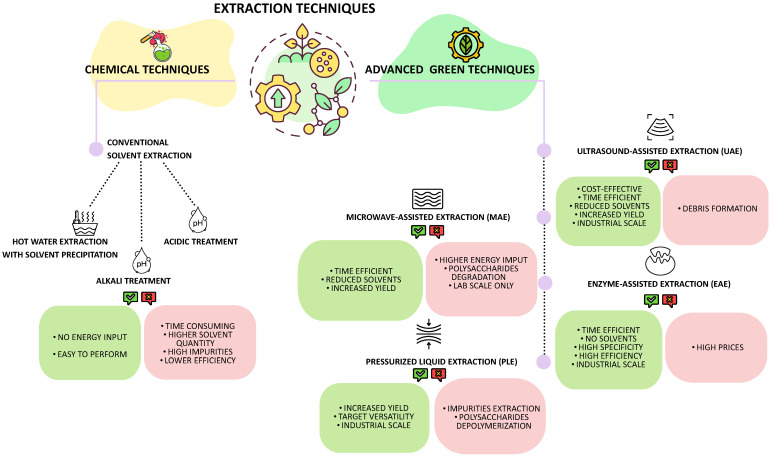
Overview of chemical and advanced green polysaccharides extraction techniques from seaweeds.

The extraction of polysaccharides from marine macroalgae is a critical step that strongly influences both yield and structural integrity. Over the years, a wide range of techniques has been developed, from conventional chemical approaches to more environmentally friendly, “green” methodologies. Each method presents specific advantages and limitations depending on the algal source, the physicochemical properties of the target polysaccharide, and the intended application. The following section provides an overview of the main extraction strategies employed for Mediterranean macroalgae *(*Figure 7).

#### 3.2.1. Chemical Techniques

Hot water extraction is usually performed on dried macroalgae, soaked at high temperature for 3 to 5 h under magnetic stirring. The supernatant is recovered after centrifugation, and alcohols (ethanol and isopropanol) are usually used to precipitate the polysaccharides from the solution. Such alcohols reduce the solubility of polysaccharides in aqueous solutions by disrupting the hydration shell surrounding the polysaccharides, resulting in their precipitation.

Although both solvents demonstrate efficacy in precipitating polysaccharides, they exhibit differences in yield and purity: isopropanol generally gives higher polysaccharide yields; however, it may concurrently precipitate undesired substances. Ethanol is preferentially utilized for its favorable safety profile and capacity to maintain superior purity levels in specific applications, notwithstanding the requirement for higher concentrations to achieve optimal yield.

The alkali extraction is commonly used to break seaweed cell walls using a basic solution such as sodium hydroxide. In this case, after extraction, the solution is neutralized.

An acidic solution, such as HCl, can penetrate algal cells. Subsequently, the H^+^ ions from the acid disrupt hydrogen bonds in polysaccharides, facilitating their release into the solvent. When it comes to targeting specific polysaccharides like Ulvans from green algae, an acidic technique is used because the polysaccharides can be better solubilized in acidic conditions [110].

Conventional solvent extractions are considered traditional methods that do not require energy input and are relatively simple to perform with readily available solvents. However, these polysaccharide extraction techniques have been demonstrated to be time-consuming, require large quantities of solvents, and yield highly impure polysaccharides with lower extraction efficiency [111,112].

#### 3.2.2. Advanced Green Techniques

Implementing green technologies in extraction processes has become imperative to address the environmental concerns and efficiency limitations associated with organic solvents. These innovative techniques are not only considered more environmentally sustainable for the reduced carbon footprint, but can also require shorter extraction times, lower temperatures, and reduced solvent volumes, resulting in improved yields and preservation of compound quality and integrity [113]. An example is given by subcritical water extraction, which utilizes water at elevated temperatures and pressures to extract polysaccharides without the need for organic solvents [7].

- Ultrasound-assisted extraction (UAE)

This technique employs ultrasonic waves that generate small vacuum bubbles, which collapse, creating cavities that can induce cell wall disruption and consequently release the compounds. Furthermore, the localized pressure and heat energy solubilize the polysaccharides [114]. Kadam, O’Donnell, et al. [115] suggested that UAE can achieve a higher extraction yield compared to chemical extraction, attributing this outcome to cavitation phenomenon. This technique demonstrates efficacy across various temperature ranges, with researchers documenting that elevated temperatures correlate with increased polysaccharide extraction yields [116,117]. Specifically, at higher temperatures, viscosity and surface tension decrease while vapor pressure increases. Consequently, this leads to the formation of more cavitation bubbles, resulting in greater cell wall disruption and, subsequently, higher yields of polysaccharides [118]. This technique cannot only be used on the industrial level but also has benefits like reducing cost and processing time, increasing yield, and faster extraction rate, and it can also be used in combination with other extraction techniques like Enzyme-Assisted Extraction (EAE) and Microwave-Assisted Extraction (MAE) [116].

An examination of sulfate content reveals no substantial disparities when compared to chemical extraction methodologies. Interestingly, the extraction of fucoidan from three brown algae species, *Nizamuddinia zanardinii* [118], *Sargassum wightii* [119], and *Ascophyllum nodosum* [112] yielded diverse sulfate levels. These levels were elevated, equivalent, and reduced, respectively, concerning those obtained through chemical extraction processes. On the other hand, the ultrasound generated by this technique can lead to chemical decomposition of the polysaccharides due to acoustic cavitation, causing hydroxyl radicals [118].

The advantages of this technique are manifold. It is a cost-effective method that utilizes a reduced quantity of solvents and is also time efficient. The polysaccharide extraction yield is significantly high, and it is a process that can be automated and applied for industrial purposes [114,120,121].

Conversely, the cavitation process during the extraction of target compounds generates fine debris from the cell wall, thereby complicating the purification process [122].

- Microwave-Assisted Extraction (MAE)

MAE is considered so far one of the most efficient extraction techniques to extract bioactive compounds from macroalgae. This methodology uses a thermal-based approach. Microwave radiation induces a rapid temperature increase and subsequent evaporation of intracellular fluids, leading to the disruption of cellular membranes and the consequent release of intracellular compounds into the extraction solvent [123]. As an extraction solvent, a solution of water with 1–2% CaCl_2_ or 0.01 M HCl can be used. The advantages of this technique are controllability, time efficiency, and energy efficiency [124]. This technique is an automated process that can potentially reduce the time required for extraction. Compared to chemical extraction, it utilizes fewer solvents and has demonstrated a higher polysaccharide yield. Extraction parameters can influence the polysaccharide’s yield due to their degradation, and it is better to adapt microwave power, irradiation time, pressure, and temperature based on the algae and the polysaccharides of interest. It has been proven that polysaccharides extracted by MAE technique can show a lower molecular weight and higher concentration of sulfate groups and consequently show a higher amount of scavenging activity compared to traditional extractions [125]. Furthermore, MAE parameters also seem to strongly influence Fucoidans from brown algae in terms of molecular weight, degree of sulfation, and monosaccharide compositions; consequently, the biological activity too [124,126].

Nevertheless, this technology has shown some disadvantages, since it requires significant energy input, and if not used properly, higher temperatures can cause degradation of the target compounds [127].

- Enzyme-Assisted Extraction (EAE)

This methodology employs specific enzymes (or a combination of them) that interact with its substrates under selected conditions, which degrade or partially degrade the algal cell wall structure, thus facilitating the release of intracellular molecules. However, the selection of appropriate polysaccharide-specific hydrolytic enzymes is required [128], and specific pH and time-temperature combination affect the rate of enzymatic reactions [129,130]. Most of these enzymes are commercially available, and the most used ones in EAE are Termamyl, Amyloglucosidase, Umamizyme, Agarase, Neutrase, Carragenanase, Alcalase, Cellucast, Ultraflo, Xylanase and Viscozyme. More specifically Celluclast is an enzyme that breaks down the algae cell wall and releases glucose, cellobiose, and longer glucose polymers; Termamyl is a heat-stable α-amylase, and Viscozyme is a mixture of carbohydrases such as hemicellulase, xylanase cellulase, and β-glucanase [128,131].

Moreover, sulfated polysaccharide yield has proven to be influenced by enzyme concentration and hydrolysis time [132].

It is common to proceed with a solvent precipitation after the EAE technique. Dore et al., 2013 [133] successfully extracted fucoidan from the brown algae *Sargassum vulgare* using the EAE technique followed by acetone precipitation; the polysaccharides showed anti-inflammatory, antioxidant, and anticoagulant effects.

This technique is considered among the more environmentally sustainable approaches, not only because the enzymes utilized are eco-friendly and non-toxic, but also because it has the potential for large-scale industrial application, potentially replacing the use of solvents. Furthermore, it demonstrated time efficiency and high specificity compared to other techniques [120,134,135]. On the other hand, the higher price of enzymes still represents an obstacle to industrial applications [123,132].

- Pressurized liquid extraction (PLE)

PLE is a novel technique that extracts compounds from macroalgae in an oxygen-free and light-free environment. It utilizes elevated temperature, which enhances sample solubility, and elevated pressure, which maintains the solvent below its boiling point. This process results in a modification of the solvent properties, enhancing its penetration and cell disruption capabilities. This technique is known by various nomenclatures depending on the extraction conditions and the solvent utilized in the process: pressurized fluid extraction (PFE), pressurized solvent extraction (PSE), accelerated solvent extraction (ASE), and subcritical water extraction (SWE) [136]. Alboofetileh et al. [116] demonstrated that the yield of fucoidan extracted with PLE from the brown algae *Nizzamuddinia zanardinii* was higher compared to chemical extraction. In addition, they were able to extract a double amount of fucoidan by using PLE from the brown algae *Saccharina japonica*, with respect to the [137,138,139,140,141,142,143,144,145,146,147,148,149]. On the other hand, when the temperature is set too high, yield could also decrease [140].

Compared to hot water extraction, MAE and EAE polysaccharides sulfate content achieved using PLE is lower; nonetheless, choosing the right solvent for the extraction might help. In fact, Saravana et al. also noted that using 0.1 M NaOH as a solvent in PLE could lead to a higher sulfate content than chemical extractions, and an acid extraction with 0.1% formic acid could lead to a lower molecular weight, probably due to the acidic solution that caused a decomposition of the polysaccharide chain [137].

Overall, this technique demonstrates high efficiency in extracting polysaccharides and exhibits commendable selectivity in targeting the desired compound. By appropriately adjusting pressure, temperature, and solvent, it is feasible to modify the target compound. Furthermore, this technique is readily applicable on an industrial scale [141]. However, elevated pressure and temperature levels may result in depolymerization and degradation of the compound. Additionally, while it is possible to target specific compounds, incorrect parameter selection may lead to the co-extraction of other compounds, such as polyphenols or pigments [141,142].

It is important to note that novel extraction technologies, such as MAE, UAE, and PLE, while representing efficient and greener alternatives to harsh chemical extraction, require careful optimization to avoid structural artifacts in seaweed-derived polysaccharides. The physical forces involved in these techniques may significantly impact polysaccharide structure, leading to partial depolymerization, modifications in monosaccharide composition, and alterations in molecular weight distribution, which can ultimately affect their biological activities [7]. In this regard, UAE has frequently been reported to induce molecular weight reduction due to cavitation-induced chain scission, particularly when high power and prolonged treatment times are applied [143]. In contrast, MAE and PLE have been demonstrated to preserve the structural integrity of sulfated polysaccharides from brown seaweeds when operated under optimized conditions. Rodríguez-Jasso et al. [124] and Yuan and Macquarrie [126] reported that MAE preserves sulfate groups and characteristic infrared fingerprints of fucoidan while limiting thermal degradation associated with prolonged conventional heating. Similarly, Saravana et al. [138] demonstrated that PLE yields fucoidan with preserved molecular weight distribution, sulfate substitution and monosaccharide composition, as further confirmed by the direct comparative study of Dobrinčić et al. [42], which showed better-preserved compositional and molecular properties for MAE- and PLE-extracted polysaccharides compared with conventional extraction. For these reasons, it is crucial that the extraction parameters of each technique are carefully optimized to minimize artifact formation based on the specific polysaccharide and seaweed matrix [144,145].

### 3.3. Purification and Characterization Techniques

Extracted polysaccharides usually exhibit contamination from proteins and low molecular weight metabolites that can persist during the extraction process. Purification techniques are usually selected based on the chemical features of the polysaccharide of interest. Commonly, chromatographic techniques, including ion-exchange chromatography, affinity chromatography, and size exclusion chromatography, are utilized.

The fundamental principle underlying each chromatographic technique involves the binding or adsorption of charged molecules to immobilize oppositely charged ion exchange groups. Sample elution is achieved by modifying the pH or concentration of the running buffer [146]. In the case of fucoidan from brown seaweed, anion exchange chromatography is the widely employed purification technique, based on the presence of sulfate groups [147,148].

Once a purified polysaccharide is obtained, the combination of chemical analysis, mass spectrometry, and Nuclear Magnetic Resonance (NMR) spectroscopy techniques are applied to obtain the molecular structure [149]. The primary structure, in terms of monosaccharide composition, is obtained after derivatization of monosaccharides into acetylated methyl glycosides (AMG) and alditol acetates (AA), analyzed by Gas Chromatography-Mass Spectrometry (GC-MS) [150,151]. Moreover, the absolute configurations of the monosaccharides (D or L configurations) are determined by chemical analysis to obtain acetylated octyl-glycosides, also analyzed by GC-MS [152]. Finally, the determination of the ring size and the glycosylation sites of the monosaccharides is obtained through the analysis of the partially methylated alditol acetates (PMAA) [153].

The determination of the primary structure is achieved through ^1^H and ^13^C homo- and heteronuclear experiments [154,155].

Finally, Infrared Spectroscopy FT-IR can provide multiple insights into polysaccharide structures, including the type of sugar rings, the presence of amino sugars, uronic acids, and sulfate groups [156]. However, infrared spectroscopy is not suitable for comprehensive structural analysis.

## 4. The Structure/Biological Activity Relationship of Mediterranean Seaweed Polysaccharides

There is increasing attention in discovering the polysaccharides’ structural features responsible for their biological activities attributable to seaweed, since the comprehension of the mechanism facilitates industrial applications.

**Figure 8:**
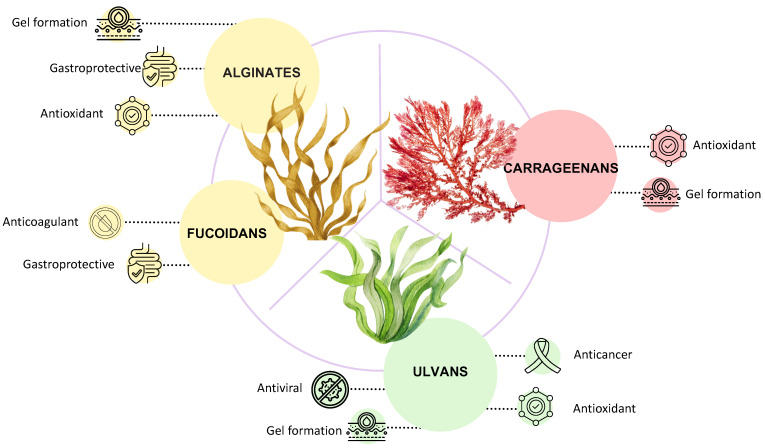
Applications and biological activities of seaweeds’ polysaccharides.

Seaweed polysaccharides are recognized to be anticancer, antimicrobial, antiviral, and anticoagulant molecules (Figure 8). Usually, as for other natural polysaccharides, their structures are strictly related to the potential activities, which can be associated with ionic bridges, hydrophobic pockets, and hydrogen bonds formation.

### 4.1. Antioxidant

Alginates from algae are frequently found to display an intense antioxidant activity, due to their capacity to scavenge the hydroxyl radical (OH^•^) and the superoxide anion radical (O_2_^•−^), the most reactive oxygen species (ROS). To detect the scavenging of these two species, two different methodologies can be used. For both, the detection consists of a final measurement of UV absorbance or EPR signal detection. The hydroxyl radical scavenging can be measured in the presence of Fe^2+^ ions in the Fenton reaction in the presence of H_2_O_2_ and salicylic acid. The hydroxylation of salicylic acid can be detected at 510 nm [67,157,158].

The superoxide anion radical is obtained when an electron is transferred to the oxygen molecule. To detect the anion, the phenazin methosulfate-NADH system has been used [159].

Two other methods to detect antioxidant activities are based on the ABTS and the DPPH reagents. The ABTS (2,2′-Azino-bis (3-ethylbenzthiazoline-6-sulfonic acid)) scavenging activity is based on the measurement of the absorbance at 734 nm, which is due to the presence of the radical ABTS^•+^. When the antioxidant is added to a solution containing ABTS^•+^ radical, the absorbance at 734 nm decreases [160]. This is an advantageous method for water-soluble molecules such as polysaccharides. Analogous reactions are the basis of the DPPH (2,2-diphenyl-1-picrylhydrazyl) methodology [160]. The reagent is not water-soluble, but the methodology is fast and reliable for those carbohydrate molecules which are soluble in organic solvents.

In many cases, to assess the antioxidant activity of a polymer, more than one of these methodologies is applied, giving in this way a real antioxidant potential of the polysaccharide [102].

One of the reasons why alginates act as antioxidants resides in the capacity of uronic acids to chelate metal ions, such as iron, and avoid the generation of radical OH [157]. Another reason explaining the activity of uronic acids containing polysaccharides can be ascribed to select abstractions of hydrogens. It has been demonstrated that the C-5 hydrogen abstraction from the uronic acid is influenced by the pH value of the solution [161]. Fucoidans are sulfated polysaccharides, and the presence of sulfates can be involved in their biological activity. It has been reported that in some cases, during the extraction of alginates and fucoidans from algae, the presence of polyphenols has been detected. Therefore, for the establishment of structure/antioxidant activity, a very pure sample of fucoidan must be obtained [28,63]. It is important to underline that in the case of Tunisian brown seaweed *Cystoseira compressa* the antioxidant activity can be attributed to the alginate and fucoidan contents since the phenolic compounds and protein amount were less than 2%, respectively [36].

Fucoidans from *Cystoseira schiffneri* revealed different antioxidant activity related to different algae collection seasons [45]. This finding could be related to the variation in structural features of the polymers, such as the molecular weight, the orientation of the sulfate groups and the monosaccharide distribution. Such correlation of structure/activity could be important for possible applications in functional food and for nutraceutical purposes.

Antioxidant activity for carrageenan has been measured by DPPH radical scavenging activity [81]. The sulfate groups may be responsible for the lowering of the energy dissociation of hydrogen bonds, thus amplifying the hydrogen-donating ability. In agreement with this observation, a positive correlation between sulfate content and antioxidant activity was found for the polysaccharides extracted from *Corallina officinalis* and *Pterocladia capillacea* [81]. Moreover, the antioxidant activity results in being augmented by the decreasing molecular weight of the polymer, since more reducing ends mean a higher capacity for blocking the radicals [162]. Therefore, the lowest is the molecular weight the highest is the activity.

Ulvan extracts from *Ulva lactuca* suggested that the antioxidant activities could be related to different structural features of the obtained polymers, such as sulfation degree, molecular weight, and monosaccharide composition [102]. Indeed, the fraction showing the lowest sulfate content and the highest Mw also indicated the highest DPPH radical scavenging activity was found for the ulvan [102]. This seems to contrast with the results reported by Yan et al., who found an increased DPPH scavenging activity and a higher content of sulfate groups after the degradation of the polysaccharide from the green seaweed *Codium cylindricum* [163].

### 4.2. Antiviral Activity

Antiviral activity has been frequently demonstrated in algae polysaccharides. In many cases, the activity has been found to depend on the presence of sulfates and their distribution [164,165]. The antiviral activity of the ulvan isolated from *Ulva lactuca* was found against HAV-10 cells and not against the *Adenovirus* [104]. In this case, authors speculated on the absorption of the polysaccharide directly on the virus or its receptors [166]. This finding is in agreement with the recently described structure/activity relationship of hydrocolloids, such as seaweed polysaccharides [166].

### 4.3. Anticancer Activity

The anticancer activity of seaweed polysaccharides can involve different mechanisms. One of these is related to the immune system since activated immune cells such as macrophages, T lymphocytes and B lymphocytes can produce various cytokines and chemokines that perform anti-tumour activity [167]. Other mechanisms are cancer cell apoptosis, inhibition of cancer cell invasion and metastasis, and scavenging free radicals [167]. Fucoidans have been reported to be involved in anticancer activity [61]. Physico-chemical properties, such as molecular weight, and chemical features, such sulfates position and degree, exert a significant impact on the ultimate biological effects of fucoidan. Fucoidans extracted from *Pavina pavonica* [41] were found to inhibit human melanoma RPMI-7951 cell line colonies. Here, the high sulfate contents of the polymers from different seasons collected seaweed samples were highlighted as the possible structure/activity relationship. The antiproliferative activity of the galactans extracted from red seaweed and tested on HeLa cells also indicated a positive dependence on the sulfate contents of the polymers [91].

Instead, in the case of ulvan from *Ulva lactuca*, the authors suggest that the molecular weight of the active polysaccharides, together with the complexity of the molecules in terms of monosaccharide composition, could be responsible for the activity [104].

### 4.4. Gastroprotective Activity

Dose-dependent gastroprotective activity has been found for alginates extracted from *Cystoseira sedoides* [38]. The alginate showed a high content M/G while displaying an MW of about 140 kDa. These physicochemical properties confirmed the relationship between the structure and the capacity to form soft and elastic gels [38].

The alginate isolated from *Dictyopteris membranaceae* and *Padina pavonica* indicated that a larger amount of uronic acids was more active on gastric ulcer induced by HCl/ethanol in rats [58]. Usually, higher viscosity has been found for longer alginate chains [168], but unfortunately, no distinct measurements of the molecular weight of the extracted polymers have been reported [28].

Highly sulfated polysaccharides may also prevent gastric damage [169]. Fucoidans from three different species of *Cystoseira* were isolated and tested in vivo for gastroprotective activity. The polymers isolated from the species *C. compressa* and *C. seidoides* showed protection against gastric mucosal lesions comparable to ranitidine, used as a reference substance. In contrast, less effect was observed for *Cystoseira crinita*. This result could be due to a slightly lower level of sulfated groups observed in this fucoidan [43].

## 5. Future Perspectives

Despite the growing interest in algal polysaccharides, research on Mediterranean macroalgae remains limited compared to other regions. Yet, the Mediterranean basin hosts a unique and diverse ecosystem in which seaweed thrives, including several endemic species that remain largely unexplored from a biochemical and biotechnological standpoint. The activities reported in this review highlight the promising Mediterranean seaweeds’ polysaccharides as sources of functional biomolecules. However, these activities have been evaluated only for a restricted number of species and often under heterogeneous experimental designs, making cross-study comparisons challenging.

From a structural standpoint, many Mediterranean seaweeds’ polysaccharides still lack high-resolution characterization. Several studies report biological activities without providing detailed structural insights or are limited to sulfation patterns, branching, or linkage analysis. In fact, NMR data are frequently incomplete or absent. Future research should prioritize full structural elucidation of Mediterranean polysaccharides, integrating advanced analytical techniques. This would enable direct comparison with well-characterized seaweeds’ polysaccharides isolated from other locations and clarify the molecular pattern that determines the observed activities, including the role of specific sulfation motifs in fucoidan gastroprotection or the influence of M/G ratios and polymer length on alginate bioactivity.

The rapid development of green extraction technologies, which are described in this study, offers sustainable and efficient alternatives to conventional chemical methods. This review highlights the strengths and limitations of both traditional chemical methods and green technologies. To overcome the intrinsic drawbacks of individual methods, such as polymer degradation, loss of sulfation, or the co-extraction of impurities, one strategy can be the implementation of combined extraction strategies, where complementary techniques are integrated into multi-step workflows to improve both yield and structural integrity [170].

Integrating these eco-friendly approaches with advanced analytical tools and standardized workflows will be key to fully unlocking the biotechnological potential of under-investigated Mediterranean macroalgal species polysaccharides and enabling their translation into high-value applications in pharmaceuticals, cosmetics, and nutraceuticals sectors.

## Figures and Tables

**Table 1:** Source, composition, and activities of polysaccharides isolated from the Mediterranean seaweeds.

	Location	Polysaccharides Yield (% *w*/*w*)	Activity/Application	Mw (g/mol)	Sulfate Content (%)	Ref.
**Brown Seaweeds**						
*Cystoseira compressa*	Kerkennah island, Tunisia	Fucoidan (5.2%) Alginate (21.65%)	Antioxidant	1 × 10^5^	Fucoidan 14.65%	[36]
*Cystoseira sedoides*	Monastir, Tunisia	Fucoidan (4.2%) Alginate (11%)	Antioxidant and Gastroprotective effect	2.8 × 10^5^ 1.4 × 10^5^	Fucoidan 15.5%	[38]
*Cystoseira barbata*	Kerkennah island, Tunisia	Laminaran (7.27%)	Antioxidant, Antibacterial Wound healing	n.d.	n.d.	[39]
*Padina pavonica*	Tunisian coast	Alginate (28.7%)	ND	4.44 × 10^6^	n.d.	[40]
*Padina pavonica*	Batroun Bay, Lebanon	Laminaran Fucoidan Alginate (seasonal variation yield)	Antitumor	n.d.	n.d	[41]
*Cystoseira barbata* *Fucus virsoides*	Zadar, Croatia Novigrad Sea, Croatia	Fucoidan 26.13% 58.55%	Antioxidant	7.66 × 10^5^–1.25 × 10^6^ 5.22 × 10^5^–8.90 × 10^5^	34.8% 25.6%	[42]
*Cystoseira crinite* *Cystoseira compressa* *Cystoseira sedoides*	Monastir and Tabarka, Tunisia	Fucoidan	Anti-inflammatory Antiradical Gastroprotective	6.42 × 10^3^ 5.45 × 10^5^ 3.39 × 10^5^	16% 16% 16%	[43]
*Cystoseira crinite*	Golf Gabes, Tunisia	Fucoidan	n.d.	n.d.	n.d.	[44]
*Cystoseira schiffneri*	Kerkennah Islands, Tunisia	Fucoidan (1–2.2%)	Antioxidant	4.0 × 10^3^–2.6 × 10^4^	7.8–34.8%	[45]
*Dictyopteris* *membranacea*	Alexandria, Egypt	n.d.	Antimicrobial Antitumor Anticoagulant	n.d.		[46]
**Red Seaweeds**						
*Pterocladia capillacea*	Alexandria, Egypt	n.d 2.8–6.46%	Antimicrobial Antitumor Anticoagulant	n.d.	n.d.	[46]
*Corallina officinalis* *Pterocladia capillacea*	Sidi Kirayr coast, Egypt	Carrageenan 37% 43%	Antioxidant antibacterial antifungal	n.d.	n.d.	[47]
*Grateloupia gibbesii Harvey*	Egypt	Agar-Type	n.d.	n.d.	n.d.	[48]
*Jania adhaerens*	Tabarka, Tunisia	Xylogalactan 4.55%	n.d.	8.0 × 10^5^	n.d.	[49]
*Galaxaulra rugosa* *Tricleocarpa fragilis* *Ligora viscida* *Osmande dechybrida* *Palisada perfosata*	Rawché beach, Lebanon	Galactans 34.8% 4.03% 10.9% 14.32% 7.5%	Antioxidant Antiproliferative	n.d.	28.5% 8.2% 25.6% 8.2% 20.3%	[50]
**Green Seaweeds**						
*Ulva lactuca*	Egypt	Ulvan	Hydrogel	n.d.	n.d.	[51]
*Ulva* *fasciata Delile*	Alexandria, Egypt	Ulvan	Antimicrobial Antifouling antioxidant Anti-inflammatory	n.d.	n.d.	[52]
*Ulva lactuca*	Monastir, Tunisia	Ulvan 11.81%	Antioxidant Scavenging activity towards DPPH radical	1.24–2.79 × 10^3^	21%	[53]
*Ulva lactuca*	Alexandria, Egypt	Ulvan	Antioxidant Antitumor	n.d.	n.d.	[54]
*Ulva lactuca* *Ulva fasciata*	Alexandria, Egypt	Ulvan 35% 37%	Antioxidant Source of C in microalgae growth	n.d.	n.d.	[55]
*Ulva lactuca*	Alexandria, Egypt	Ulvan 35%	Anticancer Antiviral Antibacterial Antioxidant	n.d.	n.d.	[56]
*Ulva rigida*	Malaga, Spain	Ulvan	Antioxidant	n.d.	n.d.	[57]

## Data Availability

No new data were created or analyzed in this study. Data sharing is not applicable to this article.

## Abbreviations

The following abbreviations are used in this manuscript:

AA	Alditol Acetates
AMG	Acetylated Methyl Glycosides
ATR	Attenuated Total Reflection
CWP	Cell Wall Polysaccharides
EAE	Enzyme-Assisted Extraction
FT-IR	Infrared Spectroscopy
GC-MS	Gas Chromatography-Mass Spectrometry
HDFs	Human Dermal Fibroblasts
HPLC	High-Performance Liquid Chromatography
MAE	Microwave-Assisted
NMR	Nuclear Magnetic Resonance
PLE	Pressurized Liquid Extraction
PMAA	Partially Methylated Alditol Acetates
ROS	Reactive Oxygen Species
SNFG	Symbol Nomenclature For Glycans
UAE	Ultrasound-Assisted Extraction
